# Surgical versus Medical Management of Progressive Familial Intrahepatic Cholestasis—Case Compilation and Review of the Literature

**DOI:** 10.3390/children10060949

**Published:** 2023-05-26

**Authors:** Maria Noelle Hüpper, Judith Pichler, Wolf-Dietrich Huber, Andreas Heilos, Rebecca Schaup, Martin Metzelder, Sophie Langer

**Affiliations:** 1 Department of Paediatric Surgery, Medical University of Vienna, 1090 Vienna, Austria; nm.huepper@gmail.com (M.N.H.);; 2 Department of Paediatric Gastroenterology, Department of Paediatrics, Medical University of Vienna, 1090 Vienna, Austriaandreas.heilos@meduniwien.ac.at (A.H.);

**Keywords:** progressive familial intrahepatic cholestasis, surgery, children, odevixibat, maralixibiat, biliary diversion, bile acid, pruritus

## Abstract

(1) Background: Progressive familial intrahepatic cholestasis (PFIC) is a rare cause of liver failure. Surgical biliary diversion (SBD) and ileal bile salt inhibitors (IBAT) can delay or prevent liver transplantation (LTX). A comparison of the two methodologies in the literature is lacking. The combination has not been investigated. (2) Methods: We performed a literature survey on medical and surgical treatments for PFIC and reviewed the charts of our patients with PFIC of a tertiary hospital. The end points of our analysis were a decrease in serum bile acid (sBA) levels, reduction of pruritus and delay or avoidance of (LTX). (3) Results: We included 17 case series on SBD with more than 5 patients and a total of 536 patients. External or internal SBD, either conventional or minimally invasive, can reduce pruritus and sBA, but not all PFIC types are suitable for SBD. Six publications described the use of two types of IBAT in PFIC with a total of 118 patients. Treatment response was dependent on genetic type and subtype. Patients with PFIC 2 (nt-BSEP) showed the best response to treatment. Four out of eleven PFIC patients underwent SBD at our centre, with two currently receiving IBAT. (4) Conclusions: Limited data on IBAT in selected patients with PFIC show safety and effectiveness, although surgical methods should still be considered as a successful bridging procedure. Further studies to evaluate a possible combination of IBAT and SBD in PFIC are warranted and treatment decision should be discussed in an interdisciplinary board.

## 1. Introduction

Progressive familial intrahepatic cholestasis (PFIC) encompasses a spectrum of rare, autosomal recessive genetic conditions that disrupt the production, transport, and secretion of bile acid (BA). A malfunction at any step of the BA secretion ultimately causes a build-up of bile acid in the intrahepatic bile ducts [1,2,3,4].

The vast majority of PFIC patients in Austria are treated at our institution, either surgically (excluding LTX) or medically. A new PFIC case is reported to our institutions roughly every two years. The live birth rate in Austria amounts to roughly 80,000 births per year [5]. Epidemiological descriptions report an estimated incidence of 1:500,000 to 1:100,000 births [1,2,4,6].

Due to the accumulation of bile acids, patients exhibit intractable pruritus, jaundice, and liver damage in the first months of life. In the past, diagnosis of PFIC relied on clinical diagnosis, exclusion of other causes of congenital cholestasis, blood tests, and liver biopsies [4]. Recently, genetic testing has become the gold standard in determining the disease as well as the genetic subtype. Three main types of PFIC are commonly identified. Rarer Types 4 to 6 have been named in the literature [3] and new genetic mutations defining subtypes of PFIC up to Type 9 are being found [7].

The standard of care to treat drug refractory pruritus and to delay LTX in PFIC was early operative biliary diversion. Before the advent of IBAT, patients typically only received anti-pruritus medication to manage their symptoms plus medication to reduce bile salts, e.g., ursodeoxycholic acid. In patients who exhibit refractory symptoms despite conservative management, SBD is a valid therapeutic option [8].

Various surgical techniques have been developed. The spectrum includes the following operative procedures:

### 1.1. Partial External Biliary Diversion (PEBD)

The PEBD is the first described bile drainage procedure by Whitington et al. in 1988 [9]. It uses a segment of the intestine as a bile duct between the gallbladder and the skin. A cholecysto-jejunal anastomosis, a side-to-side anastomosis between the jejunal ends, and a stoma outlet are created to inhibit intestinal bile acid reabsorption [8].

As a modification of this method, Metzelder et al. described the laparoscopic method of PEBD, in which the jejunal conduit and the jejunojejunostomy are constructed extracorporeally via the umbilical port incision. The side-to-end cholecysto-jejunostomy and subsequent jejuno-cutaneostomy are then created laparoscopically [10].

To avoid a stoma, Schukfeh et al. presented a modified laparoscopic procedure of PEBD using a button inserted laparoscopically directly into the gallbladder [11,12].

Another conventional surgical option is the cholecysto-appendicostomy, which was reported by Rebhandl et al. and Sharma et al. They created an anastomosis between the gallbladder and the abdominal wall using the appendix as a conduit based on the Mitrofanoff method [13,14].

### 1.2. Partial Internal Biliary Diversion (PIBD)

PIBD was developed to reduce the bile salt reabsorption in the terminal ileum without the need for an external stoma. The cholecysto-jejunocolonic (CCJC) anastomosis is one of the surgical techniques of internal biliary diversion described in the literature by Gunaydin [15]. By using a section of the jejunum as a biliary conduit between the gallbladder and the ascending colon, the bile salts can bypass the ileum. Other procedures include the cholecysto-appendicocolonic (CCAC), the cholecysto-ileocolonic (CCIC), and the cholecysto-colostomy (CCT). The latter involves connecting the colon directly to the gallbladder [16,17]. Gunaydin et al. reported seven patients with PFIC and described all four techniques of internal biliary diversion [15].

### 1.3. Ileal Exclusion (IE)

Another variant of biliary diversion without an external stoma is the exclusion of the ileum from the enterohepatic circulation. This involves cutting the continuity of the small bowel and creating an anastomosis between the proximal end of the small intestine and the ascending colon [18].

With regard to non-operative treatment, there is a clear focus in recent years on the new applicable and promising intestinal bile acid transport inhibitors (IBAT). The mechanism of action of IBAT is to block the bile acid reabsorption, reducing the bile acid volume in the general circulation. This is achieved through the inhibition of the apical sodium-dependent bile acid transporter in the terminal ileum [19]. Bile acids that are not reverted to the liver are then excreted via the faecal passage [19,20].

### 1.4. Aim of the Study

With the advent of IBATs, the current role of operative bridging procedures needs to be re-evaluated. Thus, the aim of this literature review on current case series is to compare surgical treatment options including minimally invasive procedures with the new disease-targeted medical treatments.

## 2. Materials and Methods

We compiled a list of case series on SBD with their outcomes. For our analysis, we included case reports on PubMed from January 2010 to January 2023, all of whom had PFIC as their primary diagnosis and in whom SBD was performed as a surgical intervention. Reports that contained fewer than five patients were excluded. Included case reports were analysed according to the type of PFIC, the presence of pruritus, and the method of SBD. End points were improvement of pruritus, the transplant-free survival time, the number of LTXs performed, and any complications related to the SBD or LTX. For each of the above endpoints, the number of patients was detailed in order to accurately record the outcome and compare the results between case reports.

In addition, we reviewed the literature regarding IBATs in PFIC. We included publications examining IBAT in PFIC on PubMed. We did not include studies with pending results or publications regarding the use of IBAT in cholestatic diseases other than PFIC.

Finally, we reviewed the charts of all patients with PFIC treated either conservatively or surgically at the University Clinic of Paediatric Gastroenterology and the University Clinic of Paediatric Surgery at the Medical University of Vienna in the last 21 years (2002–2023) with their endpoints of pruritus and need for LTX. In addition, we collected data on the use of IBAT in our patients as well as their initial and last documented SBA.

## 3. Results

### 3.1. Literature Review of Surgical Treatment

Baker et al. performed a systematic review and meta-analysis for publications on PFIC. They included several reports up to the year 2014. This study mainly focused on the epidemiology, clinical manifestations, quality of life, pruritus, and mortality of PFIC [1]. As PFIC is a rare disease, there are still limited information and case reports in the literature. Therefore, both scientific and clinical work need to have an overview of the current status.

Here, we provide a list of case reports from the early second decade to the present day, focusing on surgical interventions, changes in pruritus after surgery, and the period of transplant-free survival. See Table 1.

### 3.2. Literature Review of Treatment with IBATs

Six publications examine the treatment of PFIC with IBAT [7,19,20,34,35,36].

We found three case reports on the use of IBAT in patients with PFIC and three studies. See Table 2 and Table 3 for details.

The IBATs examined are Maralixibat (SHP625, “Livmarli”) and Odevixibat (A4250, “Bylvay”). Other IBATs such as Volixibat (SHP 626), Elobixibat (A3309), and Linerixibat are currently under investigation not only in the paediatric but in the adult population as well for PFIC, alagille syndrome, other causes of congenital cholestatic disorders, primary sclerosing cholangitis and chronic constipation [7,19,20,34,35,36].

### 3.3. Reduction of Pruritus in IBAT-Inhibitory Treatment

A case report in 2018 of siblings with PFIC 2 due to heterozygous missense mutation in the ABCB11 gene described the reduction of pruritus. The children received not only maralixibat but also phenylbutyrate which had been reported to increase the surface expression of the bile salt export pump [37]. After an initial drastic improvement of the pruritus along with a simultaneous reduction in serum bile salts, it recurred when the phenylbutyrate was stopped. Adjuvant anti-pruritus medication, especially oxacarbazepin, was needed initially but was gradually tapered out and finally completely discontinued six months to two years after the trial start. The children remain pruritus-free with a combination of maralixibat and phenylbutyrate [35]. Another case series reported a dramatic reduction of pruritus in concordance with bile acid reduction following treatment with Odevixibat in a patient with a novel genetic mutation that was named PFIC Type 9 [7].

Loomes et al. found that depending on the PFIC genetic subtype, reduction in pruritus (as well as a reduction in serum bile acids and improvement of quality of life) could be achieved and continuously observed in 37% of patients. Patients with PFIC and a non-truncating bile salt export pump (BSEP) mutation exhibited a better treatment response than those with a PFIC 2 and a truncating BSEP mutation or even patients with PFIC 1, i.e., FIC1 mutation [20].

Thompson et al. showed clear superiority of pruritus reduction and BA reduction of Odevixibat in comparison with a placebo [19].

### 3.4. Morbidity of IBAT Treatment

The side-effect profile of IBAT includes primarily gastrointestinal symptoms. The incidence of side effects ranges from 84 to 100% [19,20]. The majority of side effects can be classified as mild or moderate. Loomes et al. reported side effects in 100% of the patients. The majority of these were not severe and passed over time. About half of the patients exhibited fever, diarrhoea, and cough. Abdominal pain and gastroenteritis were also reported. Laboratory changes in bilirubin and levels of vitamins were also noted, which in term led to the discontinuation of the patient’s participation in the study. A singular case of recurring pancreatitis and the new appearance of liver nodules led to LTX [20]. Of all patients, 24% discontinued the study due to non-serious events in the study performed by Loomes et al. One of these patients underwent LTX [20].

The side effects reported by Thompson et al. are similar to those reported by Loomes et al., most frequently diarrhoea, fever, respiratory tract infections, vomiting, and an increase of the transaminases [19]. Additionally, an increase in pre-existing splenomegaly can be seen in some patients [19].

Generally, pre-existing elevated levels of transaminases decreased in responder groups and some non-responder groups, but increases in transaminase levels have been described in both the maralixibat and odevixibat study, either in patients with PFIC 1/FIC1 mutations, in patients with PFIC 2/BSEP mutations/responders, or in patients who additionally showed an increase in splenomegaly [19,20].

The case report of Malatack et al. did not specify any adverse events or side effects, and neither did Zhao et al. [35,36].

Of the responder groups described in the studies and the one case report, no patient underwent LTX during the observed period [7,19,20,34,35,36].

### 3.5. Mortality of IBAT Treatment

There were no deaths reported by either one of the studies [19,20,36]. Furthermore, there were no deaths reported in the timeframe of the case report which spanned a treatment duration of more than two years [36].

### 3.6. Patients from our Institution

We identified eleven patients who were treated for PFIC either by operative or medical means in the last 21 years (2002–2023).

Five patients were treated for PFIC without surgery. Four patients received SBD because of PFIC at our clinic. We also opted to include two patients who received LTX due to liver failure without undergoing SBD beforehand. Please see Table 4 for the demographics of the non-operative group and Table 5 for data of the surgical group.

Out of the ten patients treated at our hospital for PFIC, five patients were male and five female (m:f = 1:1).

In the non-surgical group, three patients were diagnosed with PFIC 3, and two were diagnosed with PFIC 4 and 2, respectively.

In the surgical group, three patients suffered from PFIC 2. One patient had PFIC 1 and one PFIC 5.

### 3.7. Reduction of Pruritus in the Non-Operative Group

Only two patients suffer from pruritus. One patient has PFIC Type 4 and the other patient has PFIC Type 2. Other symptoms include constipation, jaundice, and hepatomegaly.

### 3.8. Reduction of Pruritus in the Surgical Group

All of the patients suffered from preoperative pruritus. One child experienced complete resolution of pruritic symptoms (Patient 7). Two patients (patients 6 and 9) showed reduction immediately after surgery, but an increase in follow-up.

### 3.9. Morbidity in the Non-Operative Group

No patient in the non-operative group was given an IBAT. Except for one patient (patient 3), these patients received a combination of symptomatic medication, ursodeoxycholic acid as well as fat-soluble vitamins. Concerning laboratory changes, we found anemia, thrombocytopenia or thrombocytosis, an elevation of transaminases as well as hyperbilirubinemia. Please see Table 3 for details on serum bile salt levels.

### 3.10. Morbidity in the Surgical Group

Two patients were included first in the MARCH-PFIC-MRX 502-study (ClinicalTrials.gov Identifier: NCT03905330 and subsequently in the MRX 503-study (Clinical-Trials.gov Identifier: NCT04185363). The first patient enrolled in March 2020 and the second in October 2020. The multinational MARCH-PFIC-study investigated safety and efficacy of Maralixibat in paediatric patients with PFIC via a randomized, placebo-controlled interventional study. After the completion of this study, both patients have been continuing with Maralixibat by enrolling in the “extension study of maralixibat in patients with progressive familial intrahepatic cholestasis” study. The results of this study are expected by 2024.

### 3.11. Mortality

No patient of either group has died at the time of publication.

## 4. Discussion

The advent of bile acid transport blockers posed the question on the role and relevance of SBD techniques. However, despite the new and promising conservative treatment option via IBAT, established surgical options such as minimally invasive SBD with good outcomes must not be disregarded.

SBD, either external or internal, is generally safe and effective and can reduce the SBA. Thus, the duration of native liver survival in patients is increased and LTX due to ESLD is postponed or averted. Treatment success has been reported consistently irrespective of the various operative methods that have been developed over time. Minimally invasive methods, in particular, can offer improvement in quality of life by reducing pruritus with minimal postoperative morbidity and complication/risk profile [2,10,18]. Adverse events after SBD include stoma-related issues (i.e., peristomal skin irritation, retraction), dehydration and electrolyte imbalances in external diversion, and diarrhea and frequent bowel movements due to inciting hyperperistalsis of the colon by the bile acids [1,3,4,12,34].

Morbidity seems to be tied to the initial genetic defect and disease and is higher in PFIC1 than in PFIC2, 3, and 4 [4]. Mortality after SBD is very low and reported as being due to ESLD [18]. LTX after SBD procedures ranges from 11% in PFIC 2 to 50–75% in PFIC1, and 60% in PFIC 3, respectively. Mortality after LTX ranges from 15.6 to 37% [4].

In summary, there is compelling evidence for the effectiveness and safety of SBD making it a valuable bridging tool to reduce SBA and pruritus, improve quality of life, and avoid LTX. Even in recognition of the new non-invasive treatment option of IBAT, SBD still has its justification to be considered as valuable component of the treatment plan. Long-term data in the literature support this, while the data on IBAT and its long-term outcomes are still too scarce. 

IBAT have been developed to avoid surgical complications, by medically inhibiting the reabsorption of BA. Treatment effectiveness is measured by the reduction of pruritus with concomitant reduction of SBA, consequent improvement of the quality of life, and avoidance ofESLD with the need for LTX [19].

The effectiveness of the bile acid transport blockers seems to be tied to the underlying genetic profile of the individual patient. A deficiency of the BSEP is the cause of the most common form of PFIC, PFIC 2. The residual activity of the BSEP, even if its function is severely impaired, seems to be the key to whether the IBAT can successfully reduce the bile salt pool and therefore inhibit pruritus and normalize BA. This has been demonstrated in three case reports [7,34,35], and also in the three randomized studies conducted by Loomes et al. [20], Thompson et al. [19], and Zhao et al. [36]. The study design of Thompson et al. listed genetic mutations with complete loss of function of the BSEP as an exclusion criterion for the study [19]. Treatment success ranged from 30 to 100%. Up to 100% of patients receiving IBAT exhibit mild to moderate adverse effects [7,19,20,34,35,36].

In the patient collective of our hospital, a pattern of disease category and treatment modality can be seen in the combined patient collective of our hospital. Three patients in the non-surgical group have PFIC 3, while no patients with PFIC 3 or 4 are in the surgical group. In the non-surgical group, there is one case each of PFIC 2 and 4, whereas three patients in the operative group have PFIC 2. PFIC Type 3 and 4 were exclusively treated medically, whereas PFIC 2 was mainly treated surgically. One patient with PFIC 5 had to undergo LTX early in life. This rapid disease progression seems to be tied to this specific subtype [38]. The other patient who underwent LTX in puberty has PFIC 3; despite presenting symptoms early in life, diagnostic testing in out-of-state hospitals did not yield a definitive diagnosis, and he was genetically diagnosed only shortly before LTX.

Based on the evidence, we conclude that the new and promising medical treatment for PFIC cannot completely replace the surgical methods just yet. Different (genetic) subtypes of PFIC as well as the progress of the disease seem to warrant different treatment strategies, much like Baker et al. stated [1].

The advantages of combining the two approaches in a subset of patients have not yet been thoroughly discussed. Regarding the limited effectiveness of both medical and surgical treatment options for PFIC, the combination of the two treatment types looks promising. So far, there are no published data on specifying subsets of patients who would benefit from a combination of surgical biliary diversion and IBAT.

Therefore, medical providers need to stratify their patients with PFIC through thorough genetic testing and evaluation before discussing and embarking on a treatment plan with the patient and the family by discussion in a multidisciplinary board comprising paediatric surgeons, paediatric gastroenterologists, nurses, dietitians, and child psychologists.

## 5. Conclusions

The inhomogeneous patient population combined with the rarity of the disease presents a challenge for both paediatric gastroenterologists and paediatric surgeons in selecting different treatment modalities.

The literature suggests that medical treatment with IBAT is not effective or suitable for every patient throughout all subtypes of PFIC, just as the surgical biliary diversion shows variable effectiveness and usefulness across the subtypes. The current literature strongly supports the notion of selected patients receiving SBD dependent on their individual genetic profile, but definitive assignment is pending. The evidence for SBD as a crucial treatment module must not be overlooked, as demonstrated in the literature and in our clinical practice.

Due to the limited success of both surgical biliary diversion and pharmacological therapy using IBATs in PFIC, we have already started combining the two treatment methods at our institution.

Further examinations and studies are still needed to state the potential benefits of this treatment modality combination.

## Figures and Tables

**Table 1:** List of patient series (*n* ≥ 6) from the literature.

Author, Year, Patient Collective	Öztürk, H et al., 2022, 9 Patients [21]	Pfister, E et al., 2022, 6 Patients [3]	Mehta, S et al., 2022, 13 Patients [22]	Bjoernland, K et al. 2021, 33 Patients [23]	Li, Q et al., 2021, 41 Patients [17]	Foroutan, H et al., 2020, 44 Patients [24]	Van Vaisberg V et al., 2020, 11 Patients [18]	Erginel, B et al., 2018, 6 Patients [25]	Flores, C et al., 2018, 37 Patients [26]
PFIC type (n)	Type 1 (2) Type 2 (6) Type 3 (1)	Type 1 (1) Type 2 (1) Type 4 (1) Type 5 (1) Type 6 (2)	Type 1 (4) Type 2 (3) Type 3 (2) Type 4 (3) Type 5 (1)	Type 1 (4) Type 2 (19) Type 3 (1) Aegenaes syndrome (1) AGS (2) Unknown (6)	Type 1 (17) Type 2 (13) Type 3 (3) Low GGT syndrome (7)	Type 1 (26) Type 2 (5) Unknown type in 13 patients	Type 1 (5) Type 2 (1) Type 5 (2) ARC-syndrome (1) AGS (2)	Type 1 (1) Type 2 (2) 3 patients were not genetically diagnosed	AGS (23) Type 1 (4) Type 2 (9) Type 3 (4)
Pruritus (n/n)	Yes (9/9)	Yes (4/6)	Yes (11/13)	Yes (33/33)	Yes	Yes (44/44)	Yes (11/11)	Yes (6/6)	Yes (3/3 patients with BD)
BD (n/n)	PEBD in 9/9 patients (1 of them 1.5y after LTX)	PEBD in 1/6 patients (Type 1)	PEBD in 1 patient (Type 4) PIBD in 2 patients (Type 1)	PEBD in 33/33 patients (cholecystojejunostomy (25), button placed gall bladder (7), jejunal conduit (1))	PIBD 41/41 (CCT)	PIBD 44/44 (cholecystocolic bypass)	IE	PIBD in 6/6	PEBD (AGS (1)) PIBD (Type 1 and 2) LTX (14)
Age at BD	0.8 to 17 years (median age 4.3 years)	2 years	Unknown	0.3–13 years (median of 1.5y)	1.5 years (median age)	2 months–18 years (median of 29 m.)	2–14 years	2–5 years (mean age 3.83)	3.1 years (AGS), 2.4 years (Type 1), 3.2 years (Type 2)
Pruritus improvement	Absent in 8/9 patients	No	Temporary improvement in pruritus	Yes (absent in 10 patients (grade 1), moderate in 5 patients (grade 2), severe in 2 patients (grade 3)	Resolution of pruritus in 27/41	Yes (5D-itch scale: mean of 21.7 preop. to 5.8 postop.)	Yes (8/11)	Yes in 5/6 patients	Yes (3/3) in patients with BD
LTX (time between BD and LTX)	LTX in 4/9 patients (due to cirrhosis, HCC) (range of 3–7 years—median of 5 years)	4/6 patients received LTX, including the 1 patient with PEBD (6 months after BD); 1 time Re-LTX	6 LTX 1 LTX (Type 4) after BD (13 months after BD due to severe pruritus)	13 LTX (due to persistent pruritus), 2 patients on the waiting list for LT	LTX in 6/41 patients (median period of 1.83 years after BD)	2 LTX after BD in 2 patients	1 LTX (1 year after IE)	5/6 patients did not receive LTX	No LTX in patients with BD
Outcome	Improvements in diarrhoea, growth, LFP; no surgical complications	No improvements after BD (daily bile loss of 300–600 mL) resulting in LTX	1 patient died on waiting list for LTX due to sepsis	Early (<30 days) complications after BD (in 42%), re-operation (in 9%), stoma-related complications (in 55%)	20/41 complete resolution of symptoms, 7/41 relapse of symptoms, 13/41 ongoing symptoms	Revision surgery in 5 patients (11.3%) 13 patients became medication-free 1 patient died 4 years after PIBD on the waiting list for LT (due to cirrhosis) 1 patient died 2 weeks after BD due to complications and liver failure	1 revision-surgery 1 patient died due to end-stage liver disease (ESLD)	1 LTX 5 years after BD due to recurrence of pruritus, patient died after LTX due to sepsis (patient with Type 2)	PEBD: resolution of symptoms, no complications PIBD: initial diarrhoea, resolution of pruritus
Follow-up period	0.5–12.4 years (median of 6 years)	PEBD patient: no cholestasis/pruritus after LTX; persistent dystrophy and severe diarrhoea	1 patient died due to graft rejection; no LTX in PIBD patients during observation period	0.6–25.2 years (median of 10 years) 5 patients died (2 with BD, 1 after LTX, 1 after adhesion ileus and sepsis, 1 of unknown cause)	0.6–11.8 years (median of 4.8 years)	10–105 months (median of 54 months) 14 patients were lost to follow-up	6–109 months (mean of 60 months)	5.1–7.0 years (mean 6.1y)	12.5 years
**Author; Year; Patient collective**	**Chen, L et al. 2018, 34 patients** [27]	**Lemoine, C et al. 2017, 24 patients** [28]	**Agarwal, S et al. 2016, 24 patients** [29]	**Jankowska, I et al. 2016, 26 patients** [30]	**Ramachandran, P et al., 2014, 12 patients** [31]	**Jankowska, I et al., 2014, 9 patients** [32]	**Diao, M et al., 2013, 20 patients** [33]	**Schukfeh, N et al., 2012, 24 patients** [12]	
PFIC type (n)	Type 1 (11) Type 2 (13) Type 3 (5) Low GGT cholestasis (5)	Type 1 (10) Type 2 (13) Type 3 (1)	Type 1 (2) Type 2 (19) Type 3 (3)	Type 1 (3) Type 2 (14) No genetic testing in 9 patients	Type 1 (9) Type 2 (1) AGS (2)	Type 2 (3) No type known (6)	Type 1 (10) Type 2 (7) Type 3 (3)	Type 1 (4) Type 2 (3) Type 3 (1) No mutation analysis in 16/24 patients	
Pruritus (n/n)	Yes (34/34)	Yes	Yes (24/24)	Yes	Yes	Yes	Yes (20/20)	Yes (18/24 patients)	
BD (n/n)	PIBD in 34/34 patients (CCT)	PEBD (conversion from PEBD to PIBD in 7/24p., 5/7) (cosmetic reasons), 2/7 (electrolyte imbalances)	PIBD in 3 p. (3/3 Type 2) PEBD in 1 p.	PEBD (26/26)	PIBD (12/12)	IE (first operation in 4 p., IE converted from PEBD in 5 p.)	PIBD (20/20) (CCT)	PEBD (24/24)	
Age at BD	4–218 months (median age 19 months)	0.2–8.7 years (median age 1.3 years)	Unknown	0.4–16.6 years (median age of 2.2 years)	15–120 months (median 36 months)	0.6–21 years	10.8 months–5.11 years (median age, 1.47 years)	26 month (median age)	
Pruritus improvement	Yes 33/34	Yes (2 patients with recurrent pruritus (after conversion))	Yes	Absent in 18/24	Absent in 9/12 (75%)	Incomplete improvement in pruritus	Yes (20/20)	Yes (13/24 patients)	
LTX (time between BD and LTX)	Type 2: 1 LTX due to intractable pruritus (20 months after BD); 1 LTX due to grade IV hepatic fibrosis	8 LTX (33%) –1.6 years (0.5–3.1 years) after BD (100% Type 2 p.)	3 patients without BD received LTX due to advanced fibrosis 1 patient received LTX after BD due to intractable pruritus/fibrosis	3 LTX	1 p. received LTX due to pruritus, 2 p. were listed for LTX due to pruritus and liver failure (1 died awaiting LTX)	1 LTX after PEBD and IE (3 y. after BD)	None	LTX in 9/24 (3 p. within 1 y. after BD, 6 p. after a median of 27 m. after BD) 100% of p. with cirrhosis at time of BD received LTX, 12% of p. without cirrhosis at time of BD received LTX	
Outcome	Native liver survival rate: Type 1 (100%), Type 2 (69%), Type 3 (80%), lowGGT (100%)	transplant-free survival in 100% of Type 1 and Type 3 p.; 38% in Type 2 p.	100% of patients after BD: no progression in fibrosis and no symptoms	Improved lipid profile after BD	9 patients are asymptomatic	Good outcome in 3/9 Moderate outcome (episodes of cholestasis in 3/9 Poor outcome in 2/9 (1 LTX and 1 PEBD performed after IE)	Improvements in LFP and quality of life	Survival with native liver in 15 patients (63%) Surgical complications in 1 patient Stomal prolapse in 2 patients	
Follow up period	Unknown	Unknown	2 years	Unknown	12–48 months (median of 30 months)	3–14 years (median of 8.5 years) in pat. with primary IE 3 patients were discharged to adult medical care during observed period	12–104 months (median of 54 months)	1.6–14.3 y (median of 9.8 years)	

**Table 2:** List of case reports and series with IBAT treatment.

	Year	IBAT	No. of Patients	Pruritus Response by Type of PFIC
Malatack et al. [35]	2018	Maralixibat	2	good response/PFIC2/heterozygotic missense ABCB11
Slavetinsky and Sturm et al. [34]	2020	Odevixibat	1	good response/PFIC2/heterozygotic missense ABCB11
Pepe et al. [7]	2022	Odevixibat	1	good response/PFIC 9-homozygotic ZFYVE19/ANCHR

**Table 3:** Studies with IBAT treatment.

	Year	IBAT	No. of Patients	Pruritus Response by Type of PFIC
Loomes et al. [20]	2022	Maralixibat	33	- 37% response in PFIC2/nt-BSEP - no response in PFIC1/FIC1def - no response in PFIC2/t-BSEP
Thompson et al. [19]	2022	Odevixibat	62	- 55% response
Zhao et al. [36]	2022	Maralixibat	19	- 58% response in PFIC2/nt-BSEP

**Table 4:** Demographics of the non-operative patient group.

	Sex	PFIC Type	Genetic Mutation Profile	IBAT	First Documented sBA mmol/L	Last Documented sBA mmol/L	Initial Symptoms	Post-Treatment Symptoms
Pat. 1	M	PFIC 3	heterozygotic ABCB4	No	7/2020: 14.20	10/2022: 60.00	No pruritus, easy bruising	No pruritus
Pat. 2	F	PFIC 3	heterozygotic ABCB4	No	10/2014: 52.3	3/2023: 128.70	No pruritus, scleral icterus	No pruritus, generalised icterus
Pat. 3	F	PFIC 3	heterozygotic ABCB4	No	3/2021: 4.5	9/2021: 5.10	No pruritus, constipation	No pruritus, constipation
Pat. 4	F	PFIC 4	compound heterozygotic variants TJP-2	No	5/2020: 164.60	9/2021: 10.60	Moderate to severe pruritus	No pruritus
Pat. 5	F	PFIC 2	homozygotic ABCB 11	No	12/2021: 136.60	6/2022: 328.50	Severe pruritus	Mild to moderate pruritus

**Table 5:** Demographics of the surgical patient group.

	Sex	PFIC Type	Genetic Mutation Profile	IBAT	Surgery	Age at Surgery (Years)	First Documented sBA mmol/L	Last Documented sBA mmol/L	Initial Symptoms	Posttreatment Symptoms
Pat. 6	M	PFIC 2	Hetero-zygotic ABCB11	No	IBD, LAP cholecystocolostomy	5	6/2017: 134.90	10/2021: 75	Severe pruritus, icterus	Initial improvement, recurrence of pruritus, no icterus
Pat. 7	M	PFIC 2	tABCB111	Maralixibat	IBD, LAP cholecystocolostomy	12	10/2002: 360.2	10/2022: 33.90	Severe pruritus	Initial improvement, recurrence of moderate to severe pruritus
Pat. 8	M	PFIC 1	Homo-zygotic ATP8B1	Maralixibat	IBD, LAP cholecystocolostomy	1	10/2017: 239.20	9/2022: 99.80	Severe pruritus, icterus, emesis, light stools, and dark urine	Moderate pruritus, less icterus, normalization of stool and urine coloring
Pat. 9	F	PFIC 2	Hetero-zygotic ABCB11	No	IBD, LAP cholecystostomy, revisions	5	6/2013: 383.3	9/2022: 269.00	Severe pruritus, icterus	Severe pruritus
Pat. 10	M	PFIC 5	Homo-zygotic missense NR1H4	No	LTX	8 mth.	8/2016: 64.00	9/2022: 14.90	Icterus, decompensated acute liver failure	No pruritus
Pat. 11	M	PFIC 3	ABCB4	No	LTX	16	3/2023: 21.80	4/2023: 12.2	Icterus, decompensated acute-on-chronic liver failure	No pruritus

## Data Availability

Data is available from the authors upon reasonable request.

## Abbreviations

AGS	Alagille syndrome
BD	biliary diversion
BSEP	Bile salt export pump
CCAC	cholecystoappendicocolonic
CCIC	cholezystoileocolonic
CCJC	cholecystojejunocolonic
CCT	cholezystocolostomy
ESLD	end-stage liver disease
HCC	hepatocellular carcinoma
IBAT	ileal bile acid transporter
IBD	internal biliary diversion
IE	ileal exclusion
LAP	laparoscopic
LFP	liver function parameter
LTX	liver transplantation
PEBD	partial external biliary diversion
PIBD	partial internal biliary diversion
sBA	serum bile acids
SBD	surgical biliary diversion
SPGP	Sister of P-Glycoprotein
